# Quantification of Particle-Associated Viruses in Secondary Treated Wastewater Effluent

**DOI:** 10.1007/s12560-025-09634-6

**Published:** 2025-01-15

**Authors:** Huiyun Wu, Keegan Brighton, Jiahao Chen, Danmeng Shuai, Tiong Gim Aw

**Affiliations:** 1 Department of Environmental Health Sciences, School of Public Health and Tropical Medicine, Tulane University, 1440 Canal Street, Suite 2100, New Orleans, LA 70112 USA; 2 Department of Civil and Environmental Engineering, The George Washington University, Washington, DC USA; 3 Department of Civil and Environmental Engineering, Washington State University, Pullman, Washington USA

**Keywords:** Secondary effluents, Water reuse, Full-scale water reclamation facilities, Digital PCR, Enteric viruses, Particle sizes

## Abstract

Viruses can interact with a broad range of inorganic and organic particles in water and wastewater. These associations can protect viruses from inactivation by quenching chemical disinfectants or blocking ultraviolet light transmission, and a much higher dosage of disinfectants is required to inactivate particle-associated viruses than free viruses. There have been only few studies of the association of viruses with particles in wastewater, particularly in secondary treated effluent. As secondary effluent is the source water to the reclaimed water treatment system, this study quantified indigenous enteric viruses, and viral indicators associated with particles in secondary effluents collected from five full-scale water reclamation facilities in the United States. Particle-associated viruses were enumerated using a sequential filtration followed by microfluidic digital PCR. This study showed that enteric viruses and viral indicators (crAssphage and pepper mild mottle virus, PMMoV) were attached to particles of different sizes in secondary effluent. Significantly higher concentrations of RNA viruses including PMMoV, norovirus, and enterovirus were detected in filtrate of the sequential filtration, which contained particles < 0.45 µm. DNA viruses including adenovirus and crAssphage were found to be more associated with larger particles in secondary effluent. Overall, high correlations were observed between viral indicators and enteric viruses, supporting the use of crAssphage and PMMoV to evaluate virus removal efficiency in water and wastewater treatment processes. The association of viruses with particles in wastewater has significant implications on wastewater treatment and disinfection processes as well as virus enumeration in wastewater.

**Graphic Abstract:**

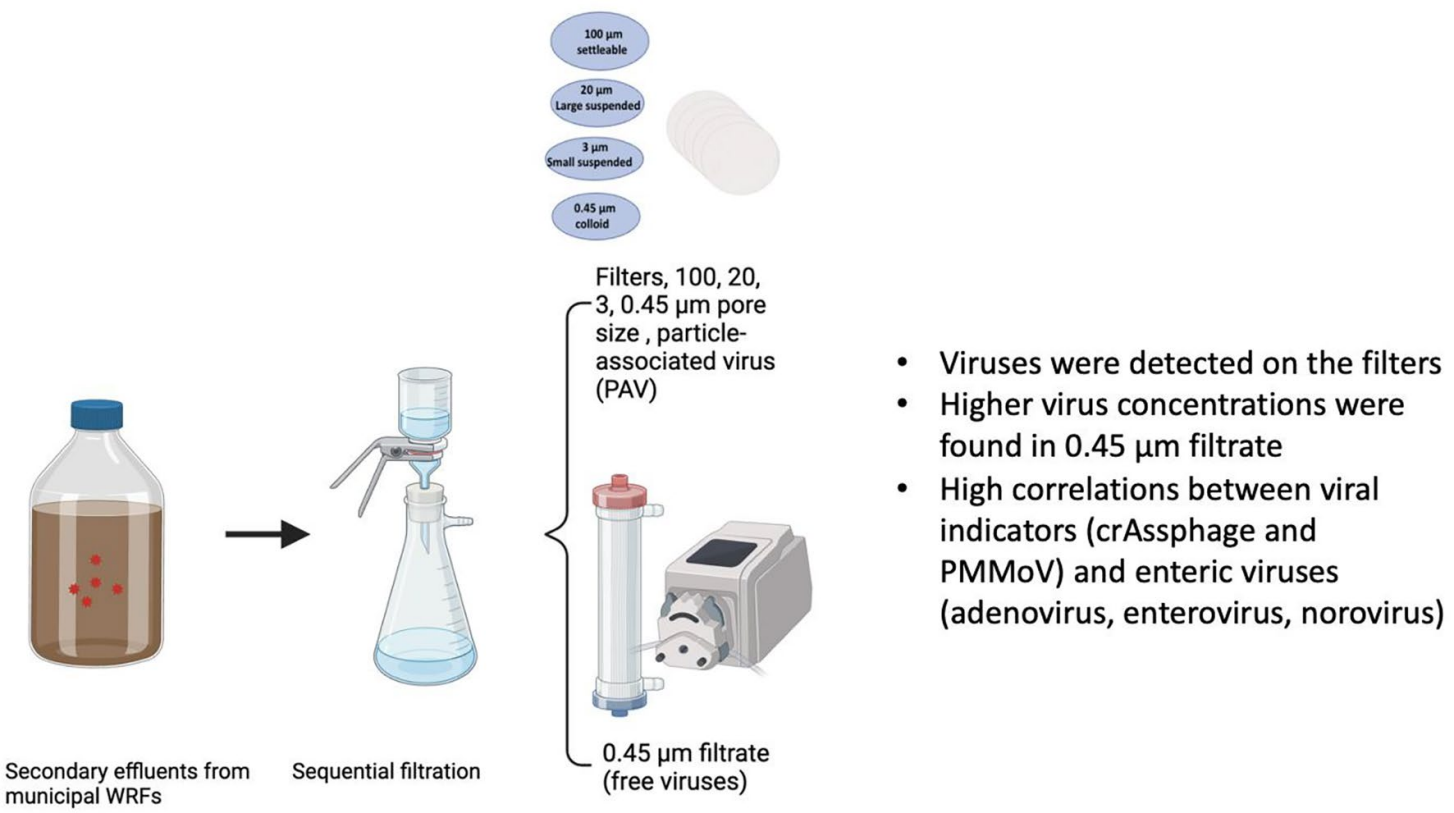

**Supplementary Information:**

The online version contains supplementary material available at 10.1007/s12560-025-09634-6.

## Introduction

Many earlier studies in environmental virology have been primarily focused on free viruses, those are not attached to particles in water and wastewater environments. Conventionally, viruses are regarded as solitary entities, transmitting and infecting hosts individually (Li et al., 2024; Schlindwein et al., 2010; Wu et al., 2023). However, in complex environments like wastewater, viruses often bind to or are associated with particles. This particle association plays a significant role in virus survival, transmission, and infectivity. Viruses can be associated with particles of sizes ranging from nanometers to millimeters in water and wastewater, including inorganic matter (e.g., minerals) (Templeton et al., 2008), organic matter (Armanious et al., 2016) such as natural organic matter (NOM), extracellular polymeric substances (EPS), microplastics, (Chahal et al., 2016; Templeton et al., 2005), and biogenic particles (e.g., algae, bacteria, protozoa, worms, and vesicles) (Atanasova et al., 2018; Zhang et al., 2022b).

Viruses can be associated with particles through electrostatic attractions and hydrophobic interactions or physical entrapment (Chahal et al., 2016; Gerba, 1984). Mechanisms driving virus’s aggregation and adsorption behaviors in water are interpreted by the extended Derjaguin, Landau, Verwey, and Overbeek (XDLVO) and steric hindrance theories. These theories consider van der Waals forces, electrostatic double layer forces, hydrophobic interactions (Wong et al., 2012), and osmotic interactions between particles (Hermansson, 1999). Hydration forces may also hinder the association of viruses with particles due to the hydration energy and have an impact on protein stability on the virus capsid (Gutierrez & Nguyen, 2012) (Verbyla & Mihelcic, 2015).

The association of viruses with particles can extend their persistence during wastewater treatment processes, necessitating higher disinfectant doses in tertiary or advanced treatment processes (Gerba & Betancourt, 2017; Templeton et al., 2008). For instance, when attached to colloid particles, viruses can shield themselves from UV exposure and direct sunlight inactivation (Templeton et al., 2005). Similarly, association with organic matter post-activated sludge treatment can protect viruses from chlorination (Hejkal et al., 1981; Verbyla & Mihelcic, 2015). Moreover, viruses associated with bacteria and protozoa can prolong their persistence and facilitate horizontal gene transfer, acting as vectors of genetic materials (Zhang et al., 2022b). Virus aggregates can further bolster virus survival in the environment and resistance to disinfectants, particularly with reactive disinfectants that can be easily consumed at the aggregate surface (Gerba & Betancourt, 2017). In specific instances, viruses associated with organic particles can enhance infection even at distant tissues or organs (Dong et al., 2023). Additionally, vesicle-cloaked virus clusters can escalate infection through *en bloc* virus transmission, evade antibody recognition, and promote infection (Kerviel et al., 2021; Zhang et al., 2022b). Therefore, the study of particle-associated viruses (PAVs) carries significant environmental, biological, and human health implications.

Treated wastewater is source of human pathogenic viruses in ambient water. Treated wastewater effluent, such as secondary effluent, often discharged into natural water bodies or recycled for various purposes in responses to water scarcity, underscores the importance of understanding pathogen prevalence. Among these pathogens, enteric viruses, particularly associated with particles, pose the highest health risk for water reuse due to their small size, low infectious dose, and high resistance to wastewater treatment processes (Gerba & Betancourt, 2019). Furthermore, understanding the prevalence and dynamics of PAVs in secondary effluent is crucial for optimizing virus removal and inactivation efficiencies in membrane-based treatment mechanisms (Dey et al., 2022).

Most of the studies on PAVs were conducted in the lab scale (Greaves et al., 2022) and often employed spiked virus surrogates (Ye et al., 2016), limiting our knowledge of indigenous viruses associated with particles in full-scale wastewater treatment facilities. This study aimed to bridge this gap by investigating particle-associated enteric viruses and viral indicators in secondary effluent which is the source for five wastewater reclamation facilities (WRFs) in the United States (U.S.). We define PAVs as those captured by filters with varying pore sizes, including 100 µm (large settleable particles), 20 µm (large suspensible particles), 3 µm (small suspensible particles), and 0.45 µm (colloid particles), with the 0.45 µm filtrate representing free viruses, vesicle viruses, and viruses associated with small particles. The objectives of this study were to (1) develop a procedure to concentrate secondary wastewater effluent for PAVs quantification using digital Polymerase Chain Reaction (dPCR), (2) identify appropriate viral indicators for PAVs, and (3) assess spatial and temporal variations of PAVs in secondary wastewater effluent.

## Method and Materials

### Secondary Effluent Sampling

From June 2022 to May 2023, a total of 32 sampling events were conducted in five WRFs in California, Florida, and Ohio (Table 1) every two weeks. The first two samples collected in June and July 2022 were used to optimize the filtration and sample processing methods, and thus were not included in the analysis. WRF A, C, D, E were sampled during winter, spring, and fall, and WRF B was sampled during all seasons. In each sampling event, 20 L of secondary effluent samples were collected, and shipped overnight in a cooler filled with ice packs. The samples were processed within 48 h after collection.

**Table 1:** Description of sampling sites

	WRF A	WRF B	WRF C	WRF D	WRF E
Location	Florida	California	California	California	Ohio
Climate zone	Humid subtropical	Hot-summer Mediterranean	Warm-summer Mediterranean	Mediterranean	Humid continental
Number of samples	6	7	8	6	5
Design capacity (MGD)	14.9	9.4	100	8	130
Average flow (MGD)	10.6	8.4	92	3–4	70
Secondary Treatment	Anoxic systems	nitrifying-denitrifying	Trickling filter/activated sludge	Activated sludge	Activated sludge w/nitrification and chemical precipitation for phosphorus removal
Tertiary or advanced Treatment	Sand filtration, chlorination	UF, 3-stage RO, UV/sodium hypochlorite	MF, RO, UV/H_2_O_2_, followed by partial decarbonation and lime stabilization	MF, RO, UV/H_2_O_2_, Product Water Storage and Blend	Seasonal chlorination (April–October)
Reuse purpose	Non-potable, aquifer recharge	IPR	IPR	Non-potable, planned IPR and DPR	Recreational, de facto reuse

Reclaimed water can be used for different purposes, ranging from indirect portable reuse (IPR) such as groundwater recharge, agricultural irrigation to direct potable reuse (DPR) as well as de facto reuse

*MF* microfiltration, *RO* reverse osmosis, *UV* UV light disinfection, *H*_*2*_*O*_*2*_ hydrogen peroxide, *UF* ultrafiltration, *IPR* indirect portable reuse, *MGD* million gallons per day

### A sequential Filtration to Isolate Particle-Associated Viruses

**Fig. 1:**
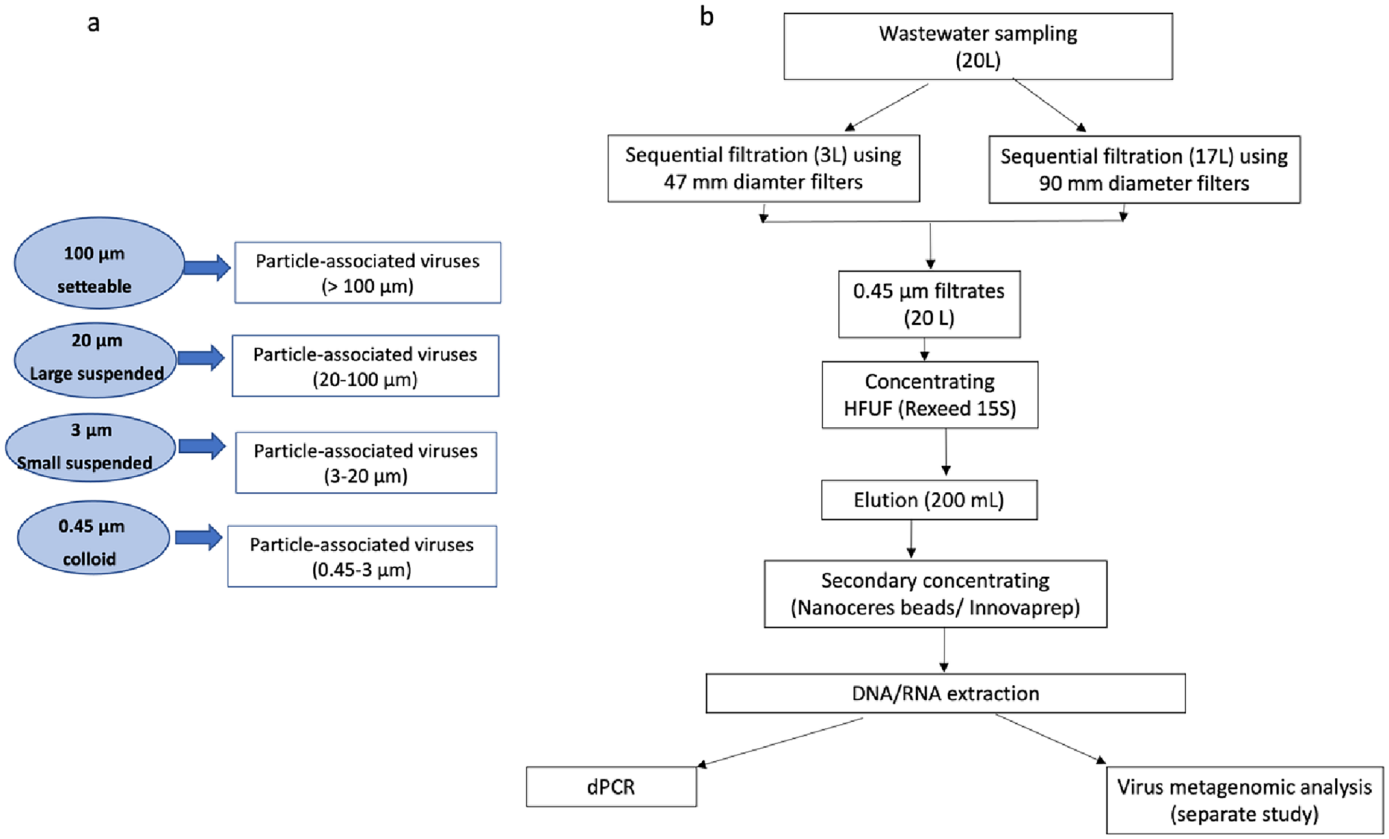
A procedure to concentrate secondary wastewater effluent for PAVs quantification. **a** A sequential filtration: secondary effluent samples were sequentially filtered through a series of four membrane filters with progressively smaller pores (100 µm, 20 µm, 3 µm, 0.45 µm) to determine the size range of particles associated with viruses. **b** A complete sample processing to analysis workflow: after sequential filtration, the 0.45 µm filtrate samples were further concentrated using hollow fiber ultrafiltration (HFUF). In this study, the 0.45 µm filtrate samples from both 47 mm diameter and 90 mm diameter filters were combined

Three liters of the secondary effluent sample were processed through 47 mm diameter membrane filters for dPCR quantification, and the rest of the sample was processed in parallel through 90 mm membrane filters for virus metagenomic analysis in a separate study (Fig. 1). Secondary effluent samples were filtered through a cascade of four membrane filters (100 µm, 20 µm, 3 µm, 0.45 µm) with sequentially smaller pores to determine the size range of particles with which viruses were associated (da Silva et al., 2008). Briefly, three liters of secondary effluent sample were filtered through a 100 µm pore size, 47 mm diameter nylon net membrane filter (Millipore sigma, Bedford MA). Filtrate of the 100 µm membrane was then filtered through a 20 µm (polycarbonate, TLD, Dawsonville, GA), a 3 µm (mixed cellulose ester, Millipore sigma, Bedford MA), and finally a 0.45 µm (mixed cellulose ester, Millipore sigma, Bedford MA) (Fig. 1a) membrane filter. The filters were kept in extraction tubes containing 0.1 mm glass beads provided by the Qiagen Allprep® Viral kit (Qiagen, Valencia, CA, USA). To avoid cake formation when filtering the samples through various pore sizes, more than one filter was used to process three liters of secondary effluent sample. Ideally, membrane filters made from the same material should be used. However, we were unable to find membrane filters made from the same material for all pore sizes during this study. Since the primary goal was to characterize particle-associated viruses based on particle size and quantify the viruses using nucleic acids extracted from the filters, filter pore size was the more critical factor. Additionally, polycarbonate, mixed cellulose ester (MCE), and nylon net membranes are hydrophobic materials that share similar properties when processing aqueous samples. To ensure effective particle size separation during sequential filtration, we monitored the vacuum pressure throughout the filtration process to prevent membrane clogging due to the cake effect.

The 0.45 µm filtrate from both 47 and 90 mm diameter membrane filters was pooled for further sample concentration (Fig. 1b). Viruses in the filtrate were concentrated using a dead-end hollow fiber ultrafilter (HFUF) with the molecular weight cut of 30 K Dalton (Rexeed 15S, Dial Medical Supply, Chester Springs, PA) using a peristaltic pump (Masterflex L/S Easy Load, Cole Parmer, Vernon Hills, IL, USA) set at 300 rpm. The ultrafilters were eluted by passing 200 mL of elution solution that contained 0.1% Tween 20, 0.01% sodium hexametaphosphate, and 0.001% Antifoam Y-30 (Sigma-Aldrich, St. Louis, MO) (McMinn et al., 2021; Wu et al., 2023). The eluate was stored in 40 mL aliquots at -80 ºC for the secondary concentration procedures using Nanotrap Microbiome A Particles® (Ceres Nanosciences, Manassas, VA) or the InnovaPrep® Concentrating Pipette Select following the manufacturer’s instruction.

Due to the time efficiency of procedure, HFUF eluate from the first 6 sampling events was processed by InnovaPrep®, and the rest of 24 sampling events were processed by Nanotrap Microbiome A Particles®. The concentration recovery efficiency from these two methods was evaluated and they were comparable. When using the InnovaPrep® Concentrating Pipette Select, 40 mL of HFUF eluate was concentrated with a 0.05 µm hollow fiber pipette tip followed by standard setting, and the eluted sample was ready for subsequent extraction. When using the Nanotrap Microbiome A Particles®, 40 mL of HFUF eluate was used for hydrogel nanoparticle binding (Longo et al., 2009). More specifically, to each sample, 100 µL of ER2 and 525 µL of Nanotrap particles were added and mixed to capture the viruses. After 10 min of room temperature incubation, the sample was placed into a magnetic rack to separate the nanoparticles from the mixture. The nanoparticles were saved and washed with 1 mL of molecular grade water, and ready for subsequent extraction. The sample and filtration information were recorded.

### Nucleic Acid Extraction

Viral nucleic acids were extracted from the filters using the Qiagen AllPrep® PowerViral Kit (Qiagen, Valencia, CA). For consistency and avoid variation due to different membrane filter pore sizes and materials, the supernatant volume after adding lysis buffer PM1 was set at 450 µL, and supernatant volume after adding IRS buffer was set at 500 µL. An extraction blank using molecular grade water was included in each extraction batch. The final elution volume of each extraction was 100 μL, which was stored as 25 μL aliquots at − 80 °C.

### Quantification of Viruses Using Digital PCR

In total, 148 samples in the form of filters (*n* = 119) and filtrate samples (*n* = 29) were used for virus quantification by the QuantStudio Absolute Q™ digital PCR system followed the MIQE guidelines (Huggett, 2020). QuantStudio Absolute Q™ is a 16-well-plate-based digital PCR (dPCR) platform with a proprietary microfluidic array plate (MAP). dPCR reactions are performed over 20,000 sub-reactions in the fixed array microchamber units. This compartmentalization capitalizes on the random distribution of nucleic acid molecules in solution, so that some of the microchambers contain single (or few) copies of target molecules and some contain none. Once the reagents have been compartmentalized into the microchambers, PCR amplification proceeds and generates a fluorescent signal in response to the presence of a target sequence.

Six viruses include cross-assembly bacteriophage (crAssphage), pepper mild mottle virus (PMMoV), FRAN coliphage Genotype I, adenovirus, enterovirus, and norovirus were quantified in single-plex assay. In the 9 µL dPCR reaction, the concentrations for primers and probe were 1 µM and 0.25 µM respectively, for all targets. The Absolute Q™ DNA digital PCR master mix (5X) and 1-step RT-dPCR master mix (4X) were used for DNA and RNA target, respectively. Each reaction well was covered with 15 µL of isolation buffer. Detailed information for the assay and dPCR cycling condition can be found in the supporting information (Tables S1 and S2).

In each dPCR plate, gblocks gene fragments (IDT, Coralville, IA, USA) were used as positive controls and nuclease-free water was used as a negative control. dPCR inhibition was tested by comparing the sample concentration with that of five-time diluted samples. Due to the low sample throughput (16 wells per plate) of the QuantStudio Absolute Q MAP plate, non-enteric viruses were mostly detected with one replicate since they were consistently in high abundance in initial plates with duplicates. dPCR quantification for adenovirus, enterovirus, and norovirus was performed in duplicate, using two wells per MAP plate for each sample.

A positive microchamber was identified by an increased fluorescent intensity compared to a negative partition, which had only a baseline signal in the same plate. The number of microchambers with successful DNA or cDNA amplification was counted, and quantification was performed by applying Poisson statistics to the proportion of the negative partitions from the total number (QuantStudio Absolute Q software Version 6) (Huggett, 2020).

### Viral Recovery Efficiency from the Filters

To determine the recovery of viruses from the membrane filters, viral nucleic acid from a homogenized secondary effluent sample collected from WRF C was extracted (200 µL) directly using the same extraction method as described in the Sect. “Nucleic Acid Extraction,” and a total of four technical replicates were performed. To assess efficiency of target capture by the filters, sequential filtrations were applied on the same samples, as it is described in the method Sect. “A sequential Filtration to Isolate Particle-Associated Viruses.” Recovery efficiencies were determined by comparing the concentration of indigenous crAssphage DNA in each sample before and after sequential filtration. We recognized differences in virus recoveries from wastewater samples between DNA and RNA viruses. The crAssphage was selected in this assessment because of its high ambient concentrations in wastewater samples.

The concentration of crAssphage from the direct extraction was compared to the sum of concentrations detected on filters with varying membrane pore sizes (membrane pore size 100 µm, 20 µm, 3 µm, and 0.45 µm) and from 0.45 µm filtrate. The mean concentration of crAssphage from the direct extraction was 2.03 × 10^6^ genome copies per liter (cp/L; standard deviation = 2.56 × 10^5^ cp/L). In contrast, the sum of the concentrations from sequential filtration was 6.01 × 10^5^ cp/L. Therefore the recovery rate of the sequential filtrations in this study was 29.64%. DNA samples extracted from the filters and filtrate were diluted fivefold to compare the concentrations with the undiluted samples, and no inhibition was found in the samples.

### Statistical Analysis

All statistics were computed using R Studio (Version 4.3.0). R code used for analysis is available on GitHub: https://github.com/wuhuiyun07/Wu_PAVanalysis_2023. Digital PCR data were log_10_ transformed and its normality was tested by the QQ-plot function. The virus targets were grouped by particle sizes, greater than 100 µm (particle retained on 100 µm pore size membrane), between 100 and 20 µm (particle retained on 20 µm pore size membrane), between 20 and 3 µm (particle retained on 3 µm pore size membrane), between 3 and 0.45 µm (particle retained on 0.45 µm pore size membrane), and less than 0.45 µm (0.45 µm pore size membrane filtrate). For spatial and temporal variation, samples were grouped into five different sites (WRF A, B, C, D, E), and the four seasons. These seasons included summer (August 2022), fall (September to November 2022), winter (December 2022 to February 2022), and spring (March to May 2023).

A diagram illustrating the dissimilarities between collected secondary effluent samples in a reduced-dimension space was created using non-metric multidimensional scaling (NMDS) based on particle sizes using the R vegan package. In this study, viral water quality indicators (crAssphage and PMMoV) and enteric viruses (adenovirus, enterovirus, norovirus) were used as variants and Bray distance between samples was calculated. A two-dimensional plot was used as the stress level from the NMDS was 0.138. An unpaired one-way ANOVA was conducted to investigate significance between mean concentrations of select pathogens in the secondary effluent samples. When appropriate, post hoc testing using Tukey’s honest significant differences test (HSD) was conducted on the log10 transformed dataset to compare the arithmetic mean concentrations of viruses sampled at five WRFs, across four seasons, and sequential filtration partitions.

The correlation structure between the viral indicators (crAssphage and PMMoV) and enteric viruses (adenovirus, enterovirus, norovirus) was evaluated using Spearman’s r coefficient (Singh et al., 2004). The analysis was performed on pooled samples from five WRFs, with a p value cutoff of 0.05. For measurements reported as non-detected or below the lower limit of measurement, the non-detected (ND) designation was replaced with half of the square root of the detection limit (0.59 gc/L) in the original dataset without log_10_ transformation (Wu et al., 2023). Non-metric multidimensional scaling (NMDS) analysis was performed on analytical composites of the enteric pathogen assays (human adenovirus, enterovirus, and norovirus). A confidence interval of 0.9 was used to delineate data ellipses (Schloss, 2023).

## Results and Discussion

### Particle-Associated Viruses Displayed Preferences on the Size of Particles in Secondary Effluents

In this study, particle size distributions of secondary effluent were determined using the SALD-2300 instrument (Shimadzu) and the size distribution was stable over time (Fig. S4). Secondary effluent samples contained a variety of particles, with sizes typically ranging from 0.2 to 0.5 µm (Table S4; Fig. S4).

**Fig. 2:**
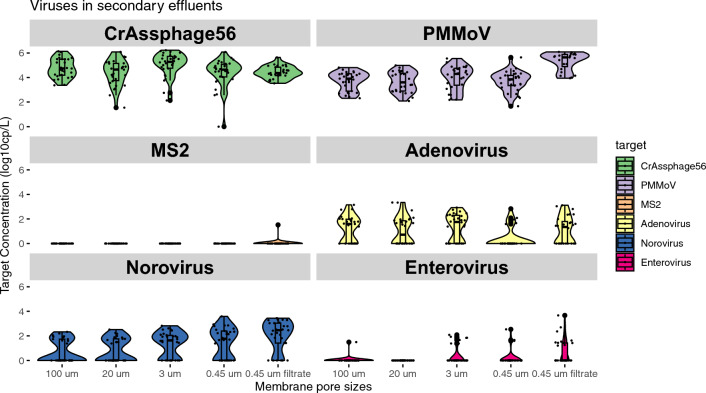
The concentrations of viruses in secondary effluents were analyzed based on particle sizes: 100 µm denotes particles larger than 100 µm, 20 µm denotes particles between 20 and 100 µm, 3 µm denotes particles 3 to 20 µm, 0.45 µm denotes particles between 0.45 and 3 µm, and 0.45 µm filtrate denotes particles smaller than 0.45 µm, which includes free viruses. Concentrations of PMMoV, enterovirus, and norovirus were significantly higher in the 0.45 µm filtrate compared to the large particle filters (*p* < 0.01). No statistically significant difference in crAssphage concentrations was found between the particles on filters and (> 0.45 µm) and in the filtrate (< 0.45 µm) (*p* = 0.25)

CrAssphage, PMMoV, and adenovirus, enterovirus, and norovirus were detected from the membrane filters with pore sizes of 100 µm (particles > 100 µm), 20 µm (particles between 20 and 100 µm), 3 µm (particles between 3 and 20 µm), and 0.45 µm (particles between 0.45 and 3 µm), and 0.45 µm filtrate (particles < 0.45 µm) (Fig. 2; Table S3). After log_10_ transformation, the PAV concentrations fit a normal distribution. RNA viruses like PMMoV, enterovirus, and norovirus were primarily detected in the 0.45 µm filtrate, while DNA viruses like crAssphage and adenovirus were found on filters, suggesting size-based particle association (Table 2). More specifically, PMMoV was detected in all filter and filtrate samples; crAssphage was found in all filter and filtrate samples, with only one 0.45 µm filter sample below the detection limit (Table 3). The highest concentrations were detected on the 3 µm filters for crAssphage and in the 0.45 µm filtrate for PMMoV, with concentrations of 1.67 × 10^6^ and 1.22 × 10^6^ gc/L, respectively (Table 3 and Fig. 2). The lowest concentrations of crAssphage and PMMoV were found on 0.45 µm filters, likely due to their lower affinity with colloid particles. CrAssphage had a significantly higher concentration (*p* < 0.05) on the 3 µm filters compared to the 0.45 µm filters, likely due to their affinity with particles like organic debris, flocs, sand, protozoa, and algae (Table S4).

**Table 2:** Characteristics of viruses of concern in this study (Heffron & Mayer, 2021)

Virus	Genome type	Isoelectric point	Genome length (kb)​	Virus capsid size (nm)	Morphology
crAssphage	dsDNA	3.8	97	77–88	Tail and icosahedral capsid
PMMoV	ssRNA	3.2	6.4	18–300	Rod
FRNA Phage	ssRNA	3.9	3.6	24	Icosahedral
Enterovirus	ssRNA	4.7	7.5	30	Icosahedral
Adenovirus	dsDNA	4.5	45	70–100	Icosahedral
Norovirus	ssRNA	5.5	7.5	27	Icosahedral

**Table 3:** Overview of virus concentrations in secondary effluents as determined by dPCR

Virus target	Number of samples (filters, filtrate)	Detection rate​	Min (cp/L)	Max (cp/L)	Mean (cp/L)	LOD (cp/L)
crAssphage56	148 (119, 29)	99.3%	0	1.67 × 10^6^	1.85 × 10^5^	15
PMMoV	137 (114, 23)	100%	45.3	1.22 × 10^6^	9.71 × 10^4^	15
MS2	94 (75, 19)	1%	0	32.4	0.35	15
Adenovirus	147 (118, 29)	49.3%	0	2.21 × 10^3^	125.1	15
Norovirus GII​	147 (118, 29)	58.8%	0	3.83 × 10^3^	230.6	15
Enterovirus	147 (118, 29)	14.2%	0	4.61 × 10^3^	43.7	15

*cp/L* genome copy per liter, *LOD* lower detection limit

Adenovirus was detected in 49.3% of the secondary effluent samples, with concentrations from below the limit of detection (< LOD) to 2.21 × 10^3^ gc/L. The highest concentrations of adenovirus were found on the 100 and 20 µm filter, indicating that adenovirus has an affinity associated with large settable and suspensible particles (Table S3). Adenoviruses, with moderate hydrophobicity due to their capsid proteins (Mangel & San Martín, 2014), interact with large particles such as sand, grit, organic debris, flocs, and protozoa, influencing their dynamics in wastewater treatment. Inorganic matter, especially sediment particles, affects the sedimentation and filtration processes, impacting pathogen removal efficiency and causing membrane fouling (Shirasaki et al., 2017; Zhang et al., 2022a). Norovirus had a higher detection rate (58.8%), with concentrations from < LOD to 3.83 × 10^3^ gc/L. Enterovirus was detected in 14.2% of samples, with concentrations from < LOD to 4.61 × 10^3^ gc/L. The highest norovirus concentrations were found on the 0.45 µm filter and in the filtrate, while the highest concentrations of enterovirus were detected in the 0.45 µm filtrate (Table 3; Fig. 2).

The adsorption of viruses on particles is influenced by the virus-particle surface interactions. The possible mechanism might also involve the surface protein property, zeta potential, and isoelectric point (pI) of viruses (Shen et al., 2011). The attachment efficiency and surface area also affect the adsorption. For example, the short tail and icosahedral capsid structure of crAssphage could facilitate its adsorption to larger particles (> 3 µm) in wastewater. Further studies are needed to elucidate the mechanisms of the particle–virus interaction in wastewater for viruses with different characteristics.

Furthermore, the hydrophobic interactions between viruses and particle surfaces may contribute significantly to virus–particle association due to the electrostatic repulsion. The salt concentration and ion strength (cation, anon, monovalent, divalent) in wastewater will affect the adsorption of viruses to particles (Gerba & Betancourt, 2017). Enteroviruses have hydrophobic pocket in capsids, which may keep them to remain suspended in water (Flatt et al., 2021). There is limited hydrophilicity information for crAssphage, while PMMoV has relatively hydrophobic capsid (Shirasaki et al., 2017). These properties are related to the electrostatic and hydrophobic interactions between the virus and particles in water systems. The hydrophobic and hydrophilic nature of viruses, along with their sizes, significantly impact their behavior and survival during wastewater treatment. Future studies should address the effects of viral characteristics on particle–virus binding mechanisms (e.g., zeta potential, isoelectric point) in wastewater to better comprehend virus fate and transport.

The association between viruses and particles is highly pH dependent (Heffron & Mayer, 2021). Different viruses have optimal pH ranges and isoelectric points to remain stable (Table 2). pH and electrical conductivity (EC) are critical parameters that influence the surface properties of viruses and affect their adsorption onto particles. Changes in pH can lead to the aggregation of viral particles or their adsorption to suspended particles. pH affects the surface charge of both virion and environmental particles (such as organic matter, minerals, and colloids), influencing the electrostatic interactions between viruses and these particles. This affects how viruses settle in and be removed during wastewater treatment process. Electrical conductivity represents the water’s ionic strength, which can influence electrostatic interactions between viruses and other particles. Higher ionic strength can potentially reducing repulsion between similarly charged particles and promoting aggregation or adsorption (Xing et al., 2020).

These water physicochemical parameters have shown to affect virus and particle surface properties. However, in real wastewater effluent samples in this study, pH and EC tend to remain relatively stable (Figure S3a). To better understand virus–particle interactions, additional water chemistry parameters, such as organic carbon concentration and total suspended solids (TSS), should be considered. It is also essential to characterize the composition of particles in wastewater, as inorganic and organic particles can interact with viruses in different ways. For instance, microplastics, an emerging contaminant of concern that is abundant in wastewater, can interact with viruses through adsorption and desorption mechanisms (Lu et al., 2022).

### Cluster Analysis of Particle-Associated Viruses

**Fig. 3:**
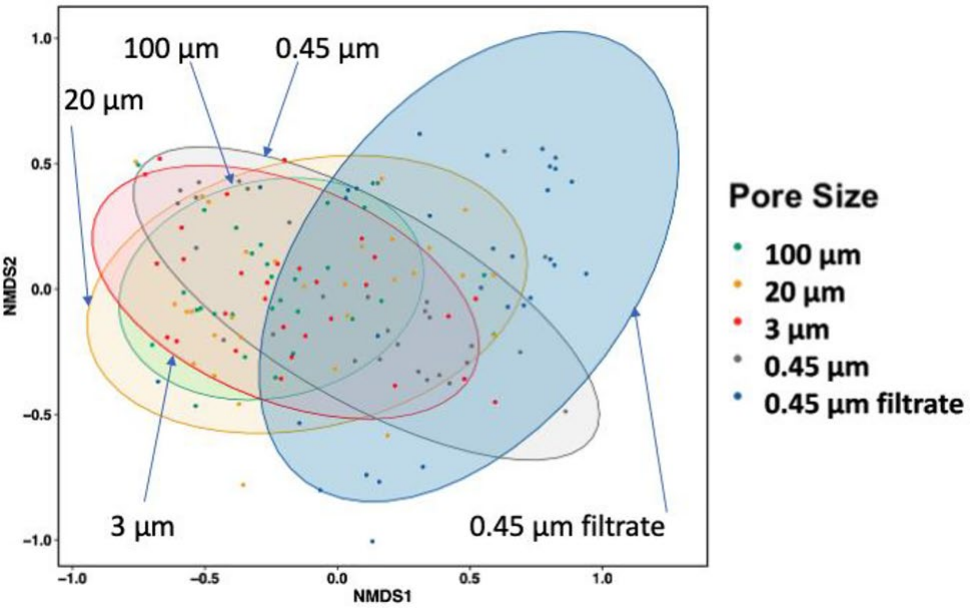
Non-Metric multidimensional scaling (NMDS) for virus communities based on particle sizes. Enteric viruses (adenovirus, enterovirus, norovirus) and viral fecal indicators (crAssphage, PMMoV) concentrations in secondary effluents were used. Sample below detection limit 15 gc/L was transformed to 0.75 gc/L. Ellipsoids represent a 90% confidence interval surrounding the data point of each particle size

Using NMDS analysis, viruses in secondary effluent samples clustered based on the particle size distributions from sequential filtration steps (Fig. 3). Virus communities overlapped between 100 and 3 µm filter samples. Virus communities associated with particles > 100 µm and < 0.45 µm were largely distinct (Fig. 3). Although viral indicators and enteric viruses were detected on large settleable particles (> 100 µm), the virus abundance and diversity were higher in 0.45 µm filtrates (with particles < 0.45 µm) (Table S3).

Using ANOVA with post hoc Tukey HSD test, the mean concentration of particle-associated crAssphage was 2.1 × 10^5^ gc/L on filters (> 0.45 µm), and 5.2 × 10^4^ gc/L in the 0.45 µm filtrate (< 0.45 µm), but this difference was not statistically significant (*p* = 0.25). The concentrations of PMMoV, enterovirus, and norovirus were significantly higher in the 0.45 µm filtrate than on the large particle filters (*p* < 0.01) (Fig. 2). Study also showed that norovirus GI and GII genogroups were more associated with particles around 0.45 µm from waste stabilization pond effluent (da Silva et al., 2008). Enteroviruses from municipal wastewater influent and unchlorinated effluent samples were found to be more associated with particles smaller than 0.3 µm (Hejkal et al., 1981). This contrasts with a previous study of primary wastewater influent that detected the highest concentrations of crAssphage, PMMoV, adenovirus, human polyomavirus, and norovirus on the 0.45 and 20 µm filters (Greaves et al., 2022). Therefore, PAVs in secondary effluents might behave differently from those in untreated wastewater, requiring special attention to PAVs on small-suspended particles and small particles (< 0.45 µm).

### Correlations Between Viral Indicators and Enteric Viruses in Secondary Effluents

**Fig. 4:**
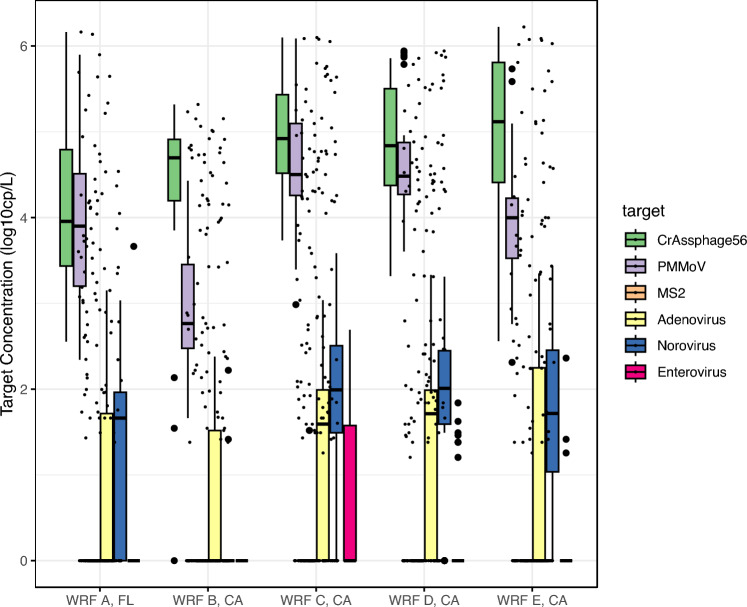
Concentrations of enteric viruses and viral indicators grouped by sampling location. Box plot with dots represents interquartile range (IQR) of the 25%, median (median line), 75% (min–max) concentrations of crAssphage (green), human adenovirus (yellow), norovirus (blue), and enterovirus (pink). The lines extending from the boxes are whiskers, which is extended to the most extreme concentration within 1.5 times IQR from the quartiles. Circles outside of the whisker range indicate outliers

Significant correlations were observed between viral indicators and enteric viruses (*p* < 0.05). Concentrations of crAssphage and PMMoV in secondary effluent samples were approximately two to four magnitudes higher than concentrations of enteric viruses (Fig. 4). Although crAssphage and PMMoV were significantly correlated with enteric viruses, we found that crAssphage had higher correlation coefficient with adenovirus (*r* = 0.64), which are DNA viruses. Such higher correlation was also found from untreated wastewater (*r* = 0.51) and from environmental water sediment (*r* = 0.37) in previous studies (Farkas et al., 2019; Wu et al., 2023). Similarly, PMMoV showed moderate correlation with RNA viruses including norovirus (*r* = 0.55) and enterovirus (*r* = 0.26) (Table 4).

**Table 4:** Spearman’s rank correlation coefficient of viral indicators and enteric viruses in secondary effluents (*n* = 148)

	CrAssphage	PMMoV	Adenovirus	Norovirus	Enterovirus
CrAssphage	1	0.19*	0.64*	0.22*	0.25*
PMMoV		1	0.15	0.55*	0.26*
Adenovirus			1	0.28*	0.35*
Norovirus				1	0.44*
Enterovirus					1

“*” Denote a significant Spearman’s rank correlation statistical significance probability* p* < 0.05

The correlation structure between the viral fecal indicators (crAssphage and PMMoV) and enteric pathogens (adenovirus, enterovirus, norovirus) was evaluated using Spearman’s r coefficient. This non-parametric measure is robust against influential data points and outliers. The analysis was performed on pooled samples from five water reclamation facilities (WRFs), with a p value cutoff of 0.05. crAssphage had higher correlation coefficient with adenovirus (*r* = 0.64), which are DNA viruses. Similarly, PMMoV showed moderate correlation with RNA viruses norovirus (*r* = 0.55) and enterovirus (0.26)

Viral indicators are often used for quantitative assessments of human fecal contamination in water environment, viral fate and transport in environmental waters, and viral removal efficiency during treatment processes (Sabar et al., 2022). Fecal bacteriophage (viruses infecting enteric bacteria), such as crAssphage and F-specific coliphage, and plant virus PMMoV have been suggested as tools for fecal source tracking, water quality monitoring, and potential surrogates to assess the performance of water and wastewater treatment processes. However, there is currently no universally accepted virus surrogate for evaluating the performance of water reuse systems. This is partly due to the diversity in virus size, shape, genome type, and viral host organisms, which result in different removal rates for viral indicators and enteric viruses (Dika et al., 2011; Gerba & Betancourt, 2017; Heffron et al., 2019). For instance, we found discrepancies between viral indicators and enteric viruses when associated with particles in secondary effluent. One concern is that while bacteriophages can adsorb to their bacterial host and propagate, enteric viruses cannot multiply outside the human host (Verbyla & Mihelcic, 2015).

In this study, MS2 phage was detected in one sample (filtrate) throughout the study (Fig. 2). MS2 phage belongs to genogroup I F-specific RNA phage (FRNA), which is commonly used as a model for enteric viruses. FRNA GI genotype has been reported as the dominant FRNA phages after wastewater treatment in Japan using plaque assay (Haramoto et al., 2012), however, similar pattern was not found in this study using dPCR assay. In this study, the PCR assay targeted only F-specific RNA phage GI genotype, whereas it could be difficult to differentiate the genotype of phages by plaque assay. When using plaque assay, the surrounding agar may have been mixed with low numbers of invisible phage plaques from other genogroup, leading to overestimate of a certain genotype. We validated the dPCR assay by using RNA from MS2 pure cultures. This study suggested that indigenous FRNA phages may have spatial variations due to the untreated wastewater characteristics and different treatment methods.

### Spatial and Temporal Distributions of PAVs in Secondary Effluent

The presence of enteric viruses has been documented in receiving waters and causing illness in communities after exposure to wastewater effluent-affected water (Rajko-Nenow et al., 2013). In this study, secondary effluent samples were collected from five full-scale WRFs in California, Ohio, and Florida, with different secondary treatment approaches (Table 1). There were statistically significant spatial differences in mean concentrations of viruses (associated with particles and free viruses) in secondary effluent by the sampling location (*p* < 0.05). Despite the geographical variations, crAssphage and PMMoV were detected in secondary effluent from all WRFs (Fig. 4). CrAssphage concentrations in secondary effluent from the WRF A were significantly lower compared to those in other WRFs (*p* < 0.001). PMMoV, adenovirus, and norovirus showed statistically significantly lower concentrations at WRF B compared to the other four WRFs, and enterovirus was not detected from WRF B (Fig. 4). These spatial variations may be due to the secondary treatment approach. Location-specific differences are impacted by in viral load, the climate zone, average wastewater influent flowrate, and serviced population. For instance, WRF B uses nitrifying-denitrifying approach as secondary treatment, which is performed in aerobic and anoxic conditions simultaneously.

Viral indicators and enteric viruses were detected throughout the seasons. CrAssphage, adenovirus, and enterovirus had no statistically significant differences in the concentration across the seasons (*p* > 0.05). CrAssphage also showed no seasonal changes in concentrations in untreated and treated wastewater, river, and seawater samples (Farkas et al., 2020). PMMoV and norovirus showed strong seasonality with significant higher concentrations in winter and spring compared to those in summer and fall (*p* < 0.01) (Figure S1). Previous studies have shown seasonal patterns of enteric viruses mainly in untreated wastewater (Wang et al., 2018; Wu et al., 2023). It is important to note that the WRF E conducts seasonal chlorination from April to October, and secondary effluents are discharged directly to a river that flows to Lake Erie during the cold months. Our finding suggested that the risk of virus-induced waterborne diseases may be higher in winter and spring compared to warmer seasons (Levy et al., 2016), as it is also supported by elevated concentrations of enteric viruses in this study. This poses significant health risks for recreational activities and limits the feasibility of potable water reuse during these periods. Additionally, the association of enteric viruses with suspended particles will dictate the fate and transport of viruses in environmental waters (Sasidharan et al., 2017), and wastewater treatment facilities could serve as point sources of viral pathogens in downstream waterbody (Prado et al., 2019b). When associated with solids, enterovirus and rotavirus retained infectious for 19 days, while their infectious only last for 9 days when freely suspended in seawater (Rao et al., 1984; Schlindwein et al., 2010).

### Implications on Water Reuse

The increasing pressure on global water supply calls for more sustainable water solutions, and various advanced treatment technologies are being investigated to provide safe water reuse to protect public health (EPA, 2012). The detection of PAVs in secondary effluents in this study has important implications on both wastewater treatment processes and virus enumeration for water reuse. In most previous studies, virus detection in wastewater samples has not counted the contribution of PAVs. A common procedure for processing water and wastewater samples for virus detection involves adsorption-extraction methods with MgCl_2_ pre-treatment and filtering the sample through 0.45 µm membrane (Ahmed et al., 2020). However, this approach may exclude viruses associated with particles smaller than 0.45 µm. The high prevalence of PAVs detected in this study suggests that PAVs should be monitored in future assessment of wastewater treatment performance for accurate estimation of virus removal. In this study, a molecular method was used to enumerate PAVs in secondary effluents, thus the viability and infectivity of these viruses were not addressed. Further studies need to determine the viability and infectivity of PAVs in wastewater using cell culture assays to better assess the viral risks for water reuse. For viruses that are difficult to culture such as norovirus, dPCR assays can be coupled with DNA/RNA intercalating dyes such as propidium monoazide (PMA) or extracellular enzymes (DNase and RNase) to distinguish potentially infectious and non-infectious PAVs (Fongaro et al., 2013).

The association of viruses with larger particles (e.g., > 100 µm) in wastewater would dictate the removal efficiency, as primary and secondary sedimentation processes are the major removal mechanisms (Chahal et al., 2016; Verbyla & Mihelcic, 2015). Viruses detected on suspended particles (20 to 3 µm), colloidal particles (3 to 0.45 µm), and particles smaller than 0.45 µm would not be removed in conventional wastewater treatment systems effectively unless tertiary treatment processes (e.g., UV or chlorine) are used or advanced treatment processes (e.g., membrane filtrations) are introduced (Table S3). Research has shown that PAVs require higher doses of disinfectants and negatively impact virus removal across different treatment approaches (Prado et al., 2019a). For example, particles smaller than 2 µm can protect viruses from UV light during disinfection (Templeton et al., 2005), and increased suspended particles challenged the virus removal during membrane bioreactor (MBR) processes (Chaudhry et al., 2015; Zhang et al., 2022a). Furthermore, viral vesicles, which are viruses clustered inside membrane-bound vesicles, present significant challenges with viral contamination and health risks due to their small sizes and resilience (Zhang et al., 2022b).

Membrane filtration methods such as microfiltration and ultrafiltration are typically used in wastewater reclamation processes (for example the WRF B, C, and D in California) to further remove suspended particles in secondary effluent (Bray et al., 2021). The viruses detected on filters in this study could be removed by membrane filtration during treatment processes, but free viruses (Fig. 3) may need further disinfection approaches such as UV for safer reuse. Alternatively, enhancing virus aggregation by electrostatic attractions could be another removal mechanism so that PAVs can be large enough to precipitate and easily separated by size (Gerba & Betancourt, 2017; Gerba et al., 2017). The inactivation of PAVs is improved when applied secondary treatments in combination with the electrochemical treatment (Chen et al., 2021; Heffron et al., 2019) and the oxidant (Gomes et al., 2019). Overall, this study provides quantitative data on PAVs in secondary effluent for better estimation of virus removal efficiency in advanced water and wastewater treatment processes.

## Supplementary Information

Below is the link to the electronic supplementary material.

Supplementary file1 (DOCX 984 KB)

## Data Availability

No datasets were generated or analyzed during the current study.

## References

[CR1] Ahmed, W., Bertsch, P. M., Bivins, A., Bibby, K., Farkas, K., Gathercole, A., Haramoto, E., Gyawali, P., Korajkic, A., McMinn, B. R., Mueller, J. F., Simpson, S. L., Smith, W. J. M., Symonds, E. M., Thomas, K. V., Verhagen, R., & Kitajima, M. (2020). Comparison of virus concentration methods for the RT-qPCR-based recovery of murine hepatitis virus, a surrogate for SARS-CoV-2 from untreated wastewater. *Science of the Total Environment,**739*, 139960. 32758945 10.1016/j.scitotenv.2020.139960 PMC7273154

[CR2] Armanious, A., Aeppli, M., Jacak, R., Refardt, D., Sigstam, T., Kohn, T., & Sander, M. (2016). Viruses at solid-water interfaces: A systematic assessment of interactions driving adsorption. *Environmental Science and Technology,**50*(2), 732–743. 26636722 10.1021/acs.est.5b04644

[CR3] Atanasova, N. D., Dey, R., Scott, C., Li, Q., Pang, X. L., & Ashbolt, N. J. (2018). Persistence of infectious enterovirus within free-living amoebae—A novel waterborne risk pathway? *Water Research,**144*, 204–214. 30031365 10.1016/j.watres.2018.07.023

[CR4] Bray, R. T., Jankowska, K., Kulbat, E., Luczkiewicz, A., & Sokolowska, A. (2021). Ultrafiltration process in disinfection and advanced treatment of tertiary treated wastewater. *Membranes (Basel),**11*(3), 221. 33804673 10.3390/membranes11030221 PMC8003589

[CR5] Chahal, C., van den Akker, B., Young, F., Franco, C., Blackbeard, J., & Monis, P. (2016). Pathogen and particle associations in wastewater: Significance and implications for treatment and disinfection processes. *Advances in Applied Microbiology,**97*, 63–119. 27926432 10.1016/bs.aambs.2016.08.001 PMC7126130

[CR6] Chaudhry, R. M., Nelson, K. L., & Drewes, J. E. (2015). Mechanisms of pathogenic virus removal in a full-scale membrane bioreactor. *Environmental Science and Technology,**49*(5), 2815–2822. 25642587 10.1021/es505332n

[CR7] Chen, M., Lei, Q., Ren, L., Li, J., Li, X., & Wang, Z. (2021). Efficacy of electrochemical membrane bioreactor for virus removal from wastewater: Performance and mechanisms. *Bioresource Technology,**330*, 124946. 33743278 10.1016/j.biortech.2021.124946

[CR8] da Silva, A. K., Le Guyader, F. S., Le Saux, J. C., Pommepuy, M., Montgomery, M. A., & Elimelech, M. (2008). Norovirus removal and particle association in a waste stabilization pond. *Environmental Science and Technology,**42*(24), 9151–9157. 19174885 10.1021/es802787v

[CR9] Dey, P., Haldar, D., Rangarajan, V., Suggala, V. S., Saji, G., & Dilip, K. J. (2022). Paradigm shift from conventional processes to advanced membrane adsorption-mediated inactivation processes towards holistic management of virus—A critical review. *Journal of Environmental Chemical Engineering,**10*(6), 108568.

[CR10] Dika, C., Duval, J. F., Ly-Chatain, H. M., Merlin, C., & Gantzer, C. (2011). Impact of internal RNA on aggregation and electrokinetics of viruses: Comparison between MS2 phage and corresponding virus-like particles. *Applied and Environment Microbiology,**77*(14), 4939–4948. 10.1128/AEM.00407-11 PMC3147388 21622784

[CR11] Dong, Z., Ma, J., Qiu, J., Ren, Q., Shan, Q. E., Duan, X., Li, G., Zuo, Y. Y., Qi, Y., & Liu, Y. (2023). Airborne fine particles drive H1N1 viruses deep into the lower respiratory tract and distant organs. *Science Advances,**9*(23), eadf2165. 37294770 10.1126/sciadv.adf2165 PMC10256160

[CR12] EPA, U.S. 2012 EPA 2012 guidelines-water-reuse, EPA National Risk Management Research Laboratory: Cincinnati, OH, USA

[CR13] Farkas, K., Adriaenssens, E. M., Walker, D. I., McDonald, J. E., Malham, S. K., & Jones, D. L. (2019). Critical evaluation of CrAssphage as a molecular marker for human-derived wastewater contamination in the aquatic environment. *Food Environ Virol,**11*(2), 113–119. 30758724 10.1007/s12560-019-09369-1 PMC6513805

[CR14] Farkas, K., Walker, D. I., Adriaenssens, E. M., McDonald, J. E., Hillary, L. S., Malham, S. K., & Jones, D. L. (2020). Viral indicators for tracking domestic wastewater contamination in the aquatic environment. *Water Research,**181*, 115926. 32417460 10.1016/j.watres.2020.115926 PMC7211501

[CR15] Flatt, J. W., Domanska, A., Seppala, A. L., & Butcher, S. J. (2021). Identification of a conserved virion-stabilizing network inside the interprotomer pocket of enteroviruses. *Commun Biol,**4*(1), 250. 33637854 10.1038/s42003-021-01779-x PMC7910612

[CR16] Fongaro, G., Nascimento, M. A., Rigotto, C., Ritterbusch, G., da Silva, A. D., Esteves, P. A., & Barardi, C. R. (2013). Evaluation and molecular characterization of human adenovirus in drinking water supplies: Viral integrity and viability assays. *Virol J,**10*, 166. 23714224 10.1186/1743-422X-10-166 PMC3686584

[CR17] Gerba, C. P. (1984). Applied and theoretical aspects of virus adsorption to surfaces. *Advances in Applied Microbiology,**30*, 133–168. 6099689 10.1016/s0065-2164(08)70054-6

[CR18] Gerba, C. P., & Betancourt, W. Q. (2017). Viral aggregation: Impact on virus behavior in the environment. *Environmental Science and Technology,**51*(13), 7318–7325. 28599109 10.1021/acs.est.6b05835

[CR19] Gerba, C. P., & Betancourt, W. Q. (2019). Assessing the occurrence of waterborne viruses in reuse systems: analytical limits and needs. *Pathogens,**8*(3), 107. 31336640 10.3390/pathogens8030107 PMC6789576

[CR20] Gerba, C. P., Betancourt, W. Q., & Kitajima, M. (2017). How much reduction of virus is needed for recycled water: A continuous changing need for assessment? *Water Research,**108*, 25–31. 27838026 10.1016/j.watres.2016.11.020 PMC7112101

[CR21] Gomes, J., Frasson, D., Quinta-Ferreira, R. M., Matos, A., & Martins, R. C. (2019). Removal of enteric pathogens from real wastewater using single and catalytic ozonation. *Water,**11*(1), 127.

[CR22] Greaves, J., North, D., & Bibby, K. (2022). Particle association and size fraction of molecular viral fecal pollution indicators in wastewater. *Environmental Science: Water Research & Technology,**8*(9), 1814–1821.

[CR23] Gutierrez, L., & Nguyen, T. H. (2012). Interactions between rotavirus and Suwannee river organic matter: Aggregation, deposition, and adhesion force measurement. *Environmental Science and Technology,**46*(16), 8705–8713. 22834686 10.1021/es301336u

[CR24] Haramoto, E., Otagiri, M., Morita, H., & Kitajima, M. (2012). Genogroup distribution of F-specific coliphages in wastewater and river water in the Kofu basin in Japan. *Letters in Applied Microbiology,**54*(4), 367–373. 22324376 10.1111/j.1472-765X.2012.03221.x

[CR25] Heffron, J., & Mayer, B. K. (2021). Virus isoelectric point estimation: Theories and methods. *Applied and Environmental Microbiology*. 10.1128/AEM.02319-20 33188001 10.1128/AEM.02319-20 PMC7848896

[CR26] Heffron, J., McDermid, B., Maher, E., McNamara, P. J., & Mayer, B. K. (2019). Mechanisms of virus mitigation and suitability of bacteriophages as surrogates in drinking water treatment by iron electrocoagulation. *Water Research,**163*, 1148788. 10.1016/j.watres.2019.114877 31349091

[CR27] Hejkal, T. W., Wellings, F. M., Lewis, A. L., & LaRock, P. A. (1981). Distribution of viruses associated with particles in waste water. *Applied and Environment Microbiology,**41*(3), 628–634. 10.1128/aem.41.3.628-634.1981 PMC243750 7224627

[CR28] Hermansson, M. (1999). The DLVO theory in microbial adhesion. *Colloids and Surfaces b: Biointerfaces,**14*(1–4), 105–119.

[CR29] Huggett, J. F. (2020). The digital MIQE guidelines update: minimum information for publication of quantitative digital PCR experiments for 2020. *Clinical Chemistry,**66*(8), 1012–1029. 32746458 10.1093/clinchem/hvaa125

[CR30] Kerviel, A., Zhang, M., & Altan-Bonnet, N. (2021). A new infectious unit: Extracellular vesicles carrying virus populations. *Annual Review of Cell and Developmental Biology,**37*, 171–197. 34270326 10.1146/annurev-cellbio-040621-032416

[CR31] Levy, K., Woster, A. P., Goldstein, R. S., & Carlton, E. J. (2016). Untangling the impacts of climate change on waterborne diseases: A systematic review of relationships between diarrheal diseases and temperature, rainfall, flooding, and drought. *Environmental Science and Technology,**50*(10), 4905–4922. 27058059 10.1021/acs.est.5b06186 PMC5468171

[CR32] Li, Y., Miyani, B., Faust, R. A., David, R. E., & Xagoraraki, I. (2024). A broad wastewater screening and clinical data surveillance for virus-related diseases in the metropolitan Detroit area in Michigan. *Human Genomics,**18*(1), 14. 38321488 10.1186/s40246-024-00581-0 PMC10845806

[CR33] Longo, C., Patanarut, A., George, T., Bishop, B., Zhou, W., Fredolini, C., Ross, M. M., Espina, V., Pellacani, G., PetricoinLiotta, E. F. L. A., & Luchini, A. (2009). Core-shell hydrogel particles harvest, concentrate and preserve labile low abundance biomarkers. *PLoS ONE,**4*(3), e4763. 19274087 10.1371/journal.pone.0004763 PMC2651577

[CR34] Lu, J., Yu, Z., Ngiam, L., & Guo, J. (2022). Microplastics as potential carriers of viruses could prolong virus survival and infectivity. *Water Research,**225*, 119115. 36137436 10.1016/j.watres.2022.119115

[CR35] Mangel, W. F., & San Martín, C. (2014). Structure, function and dynamics in adenovirus maturation. *Viruses,**6*(11), 4536–4570. 25421887 10.3390/v6114536 PMC4246237

[CR36] McMinn, B. R., Korajkic, A., Kelleher, J., Herrmann, M. P., Pemberton, A. C., Ahmed, W., Villegas, E. N., & Oshima, K. (2021). Development of a large volume concentration method for recovery of coronavirus from wastewater. *Science of the Total Environment,**774*, 145727. 33607441 10.1016/j.scitotenv.2021.145727 PMC7870434

[CR37] Prado, T., de Castro Bruni, A., Barbosa, M. R. F., Garcia, S. C., de Jesus Melo, A. M., & Sato, M. I. Z. (2019a). Performance of wastewater reclamation systems in enteric virus removal. *Science of the Total Environment,**678*, 33–42. 31075600 10.1016/j.scitotenv.2019.04.435

[CR38] Prado, T., de Castro Bruni, A., Barbosa, M. R. F., Garcia, S. C., Moreno, L. Z., & Sato, M. I. Z. (2019b). Noroviruses in raw sewage, secondary effluents and reclaimed water produced by sand-anthracite filters and membrane bioreactor/reverse osmosis system. *Science of the Total Environment,**646*, 427–437. 30056231 10.1016/j.scitotenv.2018.07.301

[CR39] Rajko-Nenow, P., Waters, A., Keaveney, S., Flannery, J., Tuite, G., Coughlan, S., O’Flaherty, V., & Dore, W. (2013). Norovirus genotypes present in oysters and in effluent from a wastewater treatment plant during the seasonal peak of infections in Ireland in 2010. *Applied and Environment Microbiology,**79*(8), 2578–2587. 10.1128/AEM.03557-12 PMC3623187 23396337

[CR40] Rao, V. C., Seidel, K. M., Goyal, S. M., Metcalf, T. G., & Melnick, J. L. (1984). Isolation of enteroviruses from water, suspended solids, and sediments from Galveston Bay: Survival of poliovirus and rotavirus adsorbed to sediments. *Applied and Environment Microbiology,**48*(2), 404–409. 10.1128/aem.48.2.404-409.1984 PMC241526 6091548

[CR41] Sabar, M. A., Honda, R., & Haramoto, E. (2022). CrAssphage as an indicator of human-fecal contamination in water environment and virus reduction in wastewater treatment. *Water Research,**221*, 118827. 35820313 10.1016/j.watres.2022.118827

[CR42] Sasidharan, S., Bradford, S. A., Šimůnek, J., Torkzaban, S., & Vanderzalm, J. (2017). Transport and fate of viruses in sediment and stormwater from a managed aquifer recharge site. *Journal of Hydrology,**555*, 724–735.

[CR43] Schlindwein, A. D., Rigotto, C., Simoes, C. M., & Barardi, C. R. (2010). Detection of enteric viruses in sewage sludge and treated wastewater effluent. *Water Science and Technology,**61*(2), 537–544. 20107281 10.2166/wst.2010.845

[CR44] Schloss, P. D. (2023). The Riffomonas YouTube channel: an educational resource to foster reproducible research practices. *Microbiology Resource Announcements,**12*(2), e0131022. 36651754 10.1128/mra.01310-22 PMC9933679

[CR45] Shen, Y., Kim, H., Tong, M., & Li, Q. (2011). Influence of solution chemistry on the deposition and detachment kinetics of RNA on silica surfaces. *Colloids and Surfaces. B, Biointerfaces,**82*(2), 443–449. 21030219 10.1016/j.colsurfb.2010.09.018

[CR46] Shirasaki, N., Matsushita, T., Matsui, Y., & Murai, K. (2017). Assessment of the efficacy of membrane filtration processes to remove human enteric viruses and the suitability of bacteriophages and a plant virus as surrogates for those viruses. *Water Research,**115*, 29–39. 28259077 10.1016/j.watres.2017.02.054

[CR47] Singh, K. P., Malik, A., Mohan, D., & Sinha, S. (2004). Multivariate statistical techniques for the evaluation of spatial and temporal variations in water quality of Gomti River (India)–a case study. *Water Research,**38*(18), 3980–3992. 15380988 10.1016/j.watres.2004.06.011

[CR48] Templeton, M. R., Andrews, R. C., & Hofmann, R. (2005). Inactivation of particle-associated viral surrogates by ultraviolet light. *Water Research,**39*(15), 3487–3500. 16081130 10.1016/j.watres.2005.06.010

[CR49] Templeton, M. R., Andrews, R. C., & Hofmann, R. (2008). Particle-associated viruses in water: Impacts on disinfection processes. *Critical Reviews in Environmental Science and Technology,**38*(3), 137–164.

[CR50] Verbyla, M. E., & Mihelcic, J. R. (2015). A review of virus removal in wastewater treatment pond systems. *Water Research,**71*, 107–124. 25613410 10.1016/j.watres.2014.12.031

[CR51] Wang, Y., Jiang, X., Liu, L., Li, B., & Zhang, T. (2018). High-resolution temporal and spatial patterns of virome in wastewater treatment systems. *Environmental Science and Technology,**52*(18), 10337–10346. 30148618 10.1021/acs.est.8b03446

[CR52] Wong, K., Mukherjee, B., Kahler, A. M., Zepp, R., & Molina, M. (2012). Influence of inorganic ions on aggregation and adsorption behaviors of human adenovirus. *Environmental Science & Technology,**46*(20), 11145–11153. 22950445 10.1021/es3028764

[CR53] Wu, H., Juel, M. A. I., Eytcheson, S., Aw, T. G., Munir, M., & Molina, M. (2023). Temporal and spatial relationships of CrAssphage and enteric viral and bacterial pathogens in wastewater in North Carolina. *Water Research,**239*, 120008. 37192571 10.1016/j.watres.2023.120008 PMC10896230

[CR54] Xing, Y., Ellis, A., Magnuson, M., & Harper, W. F., Jr. (2020). Adsorption of bacteriophage MS2 to colloids: Kinetics and particle interactions. *Colloids and Surfaces a: Physicochemical and Engineering Aspects,**585*, 1–7. 35520373 10.1016/j.colsurfa.2019.124099 PMC9067426

[CR55] Ye, Y., Ellenberg, R. M., Graham, K. E., & Wigginton, K. R. (2016). Survivability, partitioning, and recovery of enveloped viruses in untreated municipal wastewater. *Environmental Science and Technology,**50*(10), 5077–5085. 27111122 10.1021/acs.est.6b00876

[CR56] Zhang, J., Wu, B., Zhang, J., Zhai, X., Liu, Z., Yang, Q., Liu, H., Hou, Z., Sano, D., & Chen, R. (2022a). Virus removal during sewage treatment by anaerobic membrane bioreactor (AnMBR): The role of membrane fouling. *Water Research,**211*, 118055. 35042072 10.1016/j.watres.2022.118055

[CR57] Zhang, M., Altan-Bonnet, N., Shen, Y., & Shuai, D. (2022b). Waterborne human pathogenic viruses in complex microbial communities: Environmental implication on virus infectivity, persistence, and disinfection. *Environmental Science and Technology,**56*(9), 5381–5389. 35434991 10.1021/acs.est.2c00233 PMC9073700

