# Supplementary material for: Quantification of Particle-Associated Viruses in Secondary Treated Wastewater Effluent
Source: Food Environ Virol. 2025 Jan 15;17(1):19. doi: 10.1007/s12560-025-09634-6 (PMC11735553; doi:10.1007/s12560-025-09634-6)
Supplement: Supplementary file 1 — Supplementary file1 (DOCX 984 KB) [file 12560_2025_9634_MOESM1_ESM.docx]

**Supplementary Material**

**Quantification of Particle-associated Viruses in Secondary Treated Wastewater Effluent** Huiyun Wu^1,3^, Keegan Brighton^1^, Jiahao Chen^2^, Danmeng Shuai^2^, Tiong Gim Aw^1^*

^1^Department of Environmental Health Sciences, School of Public Health and Tropical Medicine, Tulane University, New Orleans, Louisiana

^2^Department of Civil and Environmental Engineering, The George Washington University, Washington, DC

^3^Department of Civil and Environmental Engineering, Washington State University, Pullman, Washington

*Corresponding author:

Tiong Gim Aw

Mailing address:

Department of Environmental Health Sciences

School of Public Health and Tropical Medicine

Tulane University

1440 Canal Street, Suite 2100

New Orleans, LA 70112, USA

Phone: +1 504-988-9926

Email: [taw@tulane.edu](mailto:taw@tulane.edu)

**Table S1.** dPCR assays

| **Assay** | **Target** | **Primer/probe** | **Sequence (5’->3’)** | **Annealing Temp (℃)** | **Reference** |
| --- | --- | --- | --- | --- | --- |
| CrAssphage | CPQ_056 | 056F1 | CAGAAGTACAAACTCCTAAAAAACGTAGAG | 60 | ^1^ |
|  |  | 056R1 | GATGACCAATAAACAAGCCATTAGC |  |  |
|  |  | 056P1 | FAM- AATAACGATTTACGTGATGTAAC -MGB |  |  |
| PMMoV | pmmov | PMMV-FP | GAG TGG TTT GAC CTT AAC GTT GA |  |  |
|  |  | PMMV-RP | TTG TCG GTT GCA ATG CAA GT | 53 | ^2^ |
|  |  | PMMV-Probe | FAM-CCT ACC GAA GCA AAT G-BHQ |  |  |
| adenovirus | JTVX | JTVXF | GGACGCCTCGGAGTACCTGAG | 55 | ^3^ |
|  |  | JTVXR | ACIGTGGGGTTTCTGAACTTGTT |  |  |
|  |  | JTVXP | FAM-CTGGTGCAGTTCGCCCGTGCCA-BHQ |  |  |
| enterovirus | EQ | EQ-1 | ACATGGTGTGAAGAGTCTATTGAGCT |  | ^4^ |
|  |  | EQ-2 | CCAAAGTAGTCGGTTCCGC | 60 |  |
|  |  | EQ-P | FAM-TCCGGCCCCTGAATGCGGCTAAT-TAMRA |  |  |
| norovirus | GII | Forward | CARGARBCNATGTTYAGRTGGATGAG | 60 | ^5^ |
|  |  | Backward | TCGACGCCATCTTCATTCACA |  |  |
|  |  | Probe | FAM-TGGGAGGGCGATCGCAATCT-BHQ |  |  |
| MS2 | MS21 | MS2 1 for | GTCCATACCTTAGATGCGTTAGC |  | ^6^ |
|  |  | MS2 1 rev | CCGTTAGCGAAGTTGCTTGG | 55 |  |
|  |  | MS2 1 probe | FAM-ACGTCGCCAGTTCCGCCATTGTCG-BHQ |  |  |

**Table S2.** dPCR cycling conditions.

| Assay | dPCR conditions |
| --- | --- |
| crAssphage | 95 ºC for 10 mins, followed by 45 cycles of 95 ºC for 3s and 60 ºC for 15s. |
| adenovirus | 95 ºC for 10 mins, followed by 40 cycles of 95 ºC for 3s, 55 ºC for 5 s and 72ºC for 3s |
| PMMoV | 55ºC for 10 min, 95 ºC for 10 mins, followed by 40 cycles of 96ºC for 5s and 60ºC for 10 s |
| enterovirus | 55ºC for 10 min, 95 ºC for 10 mins, followed by 40 cycles of 96ºC for 5s and 60ºC for 10 s |
| norovirus | 55ºC for 10 min, 95 ºC for 10 mins, followed by 40 cycles of 96ºC for 5s and 60ºC for 10 s |
| MS2 | 55ºC for 10 min, 95 ºC for 10 mins, followed by 40 cycles of 95ºC for 5s and 55ºC for 20 s. |

**Table S3.** Characteristics of viral indicators and enteric viruses detected from membrane filters and filtrate.

| **Filter pore sizes (number of samples)​** | **gc/L (min to max)​** |
| --- | --- |
| ​  100 µm filters (29)​ | crAssphage (2.32 × 10^3^ to 1.37 × 10^6^)​  PMMoV (204 to 6.42 × 10^4^)​  adenovirus (0 to 1.41 × 10^3^)​  enterovirus (0 to 30)​  norovirus (0 to 212)​ |
| ​  20 µm filters (30)​ | crAssphage (34 to 1.22 × 10^6^)​  PMMoV (121 to 9.68 × 10^4^)​  adenovirus (0 to 2.21 × 10^3^)​  norovirus (0 to 323)​  enterovirus (0 to 0)​ |
| 3 µm filters (30)​ | crAssphage (135 to 1.67 × 10^6^)​  PMMoV (151 to 3.53 × 10^5^)​  adenovirus (0 to 839)​  norovirus (0 to 664)​  enterovirus (0 to 120)​ |
| ​  0.45 µm filters (30)​ | crAssphage (0 to 1.20 × 10^6^)​  PMMoV (45.3 to 4.34 × 10^5^)​  adenovirus (0 to 678)​  norovirus (0 to 3.83 × 10^3^)​  enterovirus (0 to 340)​ |
| ​  0.45 µm filtrate (29)​ | crAssphage (3.36 × 10^3^ to 4.37 × 10^5^)​  PMMoV (8.84 × 10^3^ to 1.22 × 10^6^)​  adenovirus (0 to 1.30 × 10^3^)​  norovirus (0 to 2.74 × 10^3^)​  enterovirus (0 to 4.61 × 10^3^)​ |

**Table S4.** Particles in secondary treated wastewater

| **Particles in secondary treated wastewater^7^** | | | | |
| --- | --- | --- | --- | --- |
| Particle size (µm) | Category | Organic particles | Inorganic particles | Biogenic particles |
| >100 | large settleable particles, | organic debris, flocs, microplastics | sand and grit | Sewage warms (e.g. Tubifex tubifex) |
| 20 to 100 | large suspensible particles | organic debris, flocs, microplastics | sand and grit | protozoa |
| 3 to 20 | small suspensible particles | organic debris, flocs, microplastics | sand | protozoa, algae |
| 0.45 to 3 | colloid particles | organic debris, flocs, microplastics | inorganic precipitates (silica) | bacteria |
| <0.45 | small particles | cell fragments, proteins, carbohydrates, fatty acid, vitamins, amino acid, nanoplastic, EPS | metal Ions (e.g. heavy metals); nutrients, trace contaminants (PFAS, pharmaceuticals, personal care products) | vesicles, viruses |

**Figure S1.** Temporal variation of the particle associated viruses and free viruses in secondary effluent.

**Figure S2.** Viral concentration by extraction type in secondary effluent. Filter denotes all filters from various membrane pore size pooled samples, filtrate denotes 0.45 µm filtrate.

**
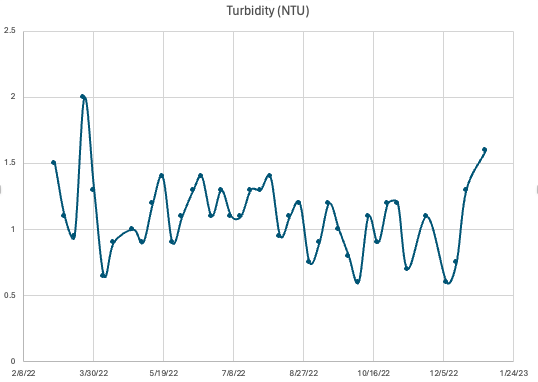

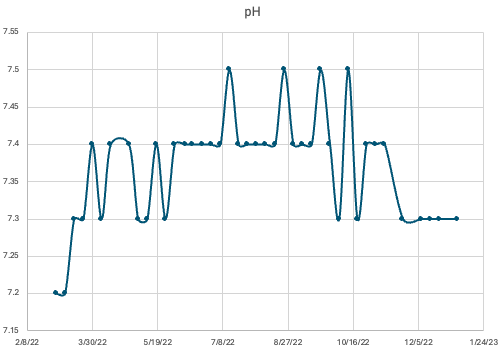
**

**
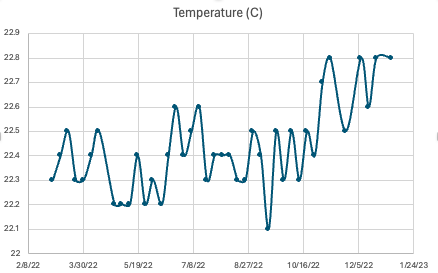

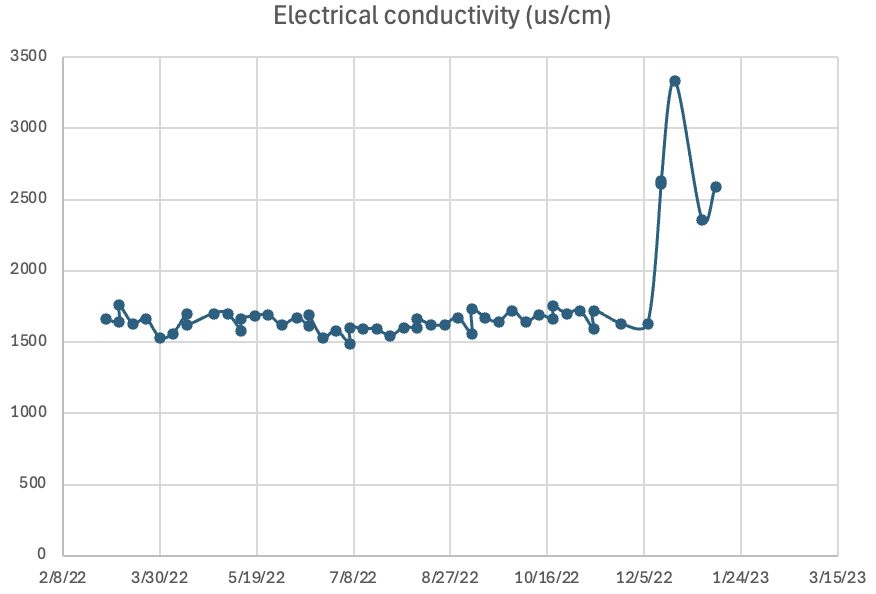
**

**Figure S3a.** Physical chemical parameters from WRF C, CA.


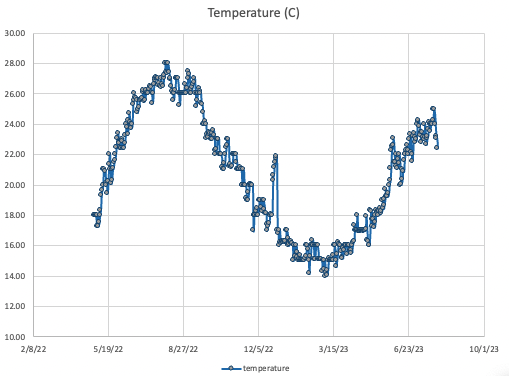

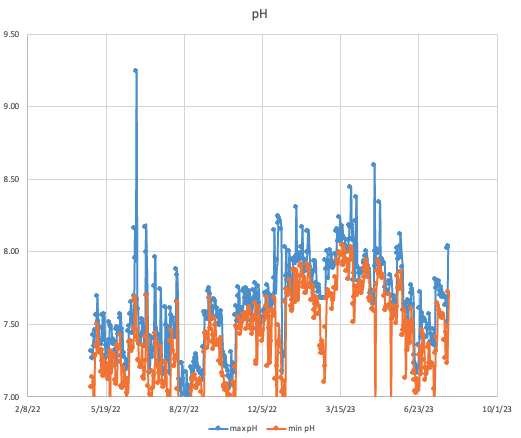


**Figure S3b.** Physical chemical parameters from WRF E, OH.

## **Particle size distribution analysis methods**

The hydrodynamic size distribution and concentration were determined using the SALD-2300 instrument (Shimadzu) following to the manufacturer’s protocols. Prior to and between each sample measurement, ultrapure water was employed to verify the background signal and cleanse the batch cell unit. A volume of 200 mL of each sample was introduced into the batch cell unit and circulated for 1 minute to ensure thorough mixing. Manual mode was utilized during the measurement process, with consistent settings maintained across all samples to enable reliable comparison of results. These settings included circulation at level 7, signal accumulation count of 2, signal averaging count of 64, and an interval of 60 seconds. Additionally, the absorbance range (0.01 – 0.2) and particle size range (0.017 – 2500 µm) were standardized. The cumulative percentage (Q3) of particles below defined micron sizes and the differential distribution (q3) at defined micron sizes were shown in Figure S4.

**
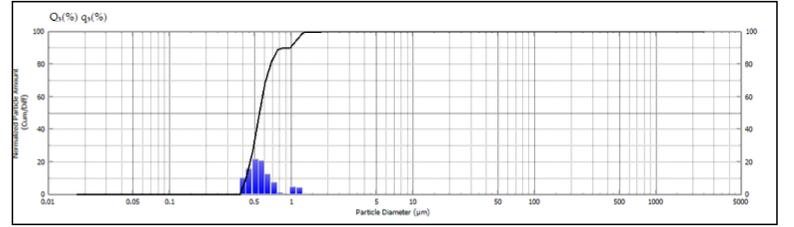
**

WRF A, Florida


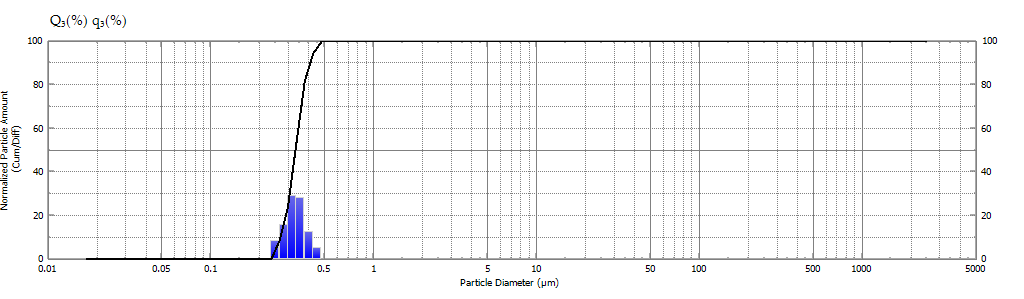


WRF B, California

**
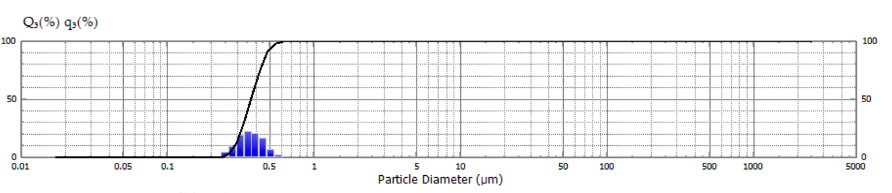
**

WRF C, California


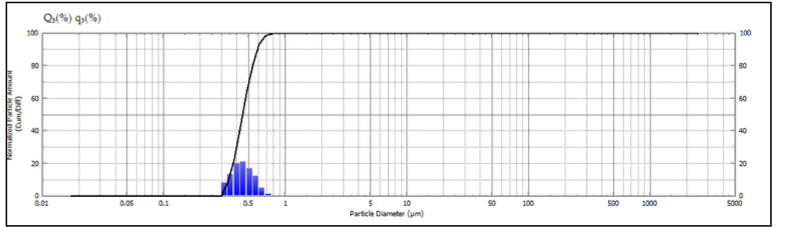


WRF D, California

**
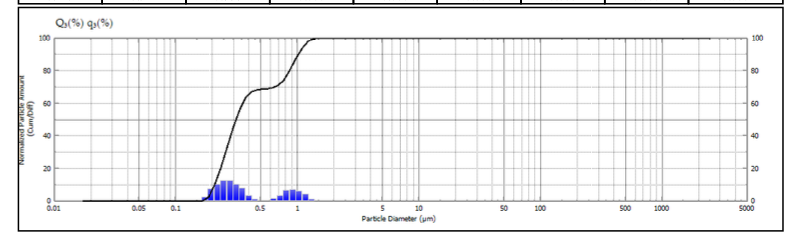
**

WRF E, Ohio

**Figure S4.** Particle size distributions in secondary effluent samples as measured by Particle Size Analyzer (laser)

**R code available on GitHub:**

https://github.com/wuhuiyun07/Wu_PAVanalysis_2023

**References**

(1) Stachler, E.; Kelty, C.; Sivaganesan, M.; Li, X.; Bibby, K.; Shanks, O. C. Quantitative CrAssphage PCR Assays for Human Fecal Pollution Measurement. *Environ Sci Technol* **2017**, *51* (16), 9146-9154. DOI: 10.1021/acs.est.7b02703.

(2) Rosario, K.; Symonds, E. M.; Sinigalliano, C.; Stewart, J.; Breitbart, M. Pepper mild mottle virus as an indicator of fecal pollution. *Appl Environ Microbiol* **2009**, *75* (22), 7261-7267. DOI: 10.1128/aem.00410-09 From NLM.

(3) Jothikumar, N.; Cromeans, T. L.; Hill, V. R.; Lu, X.; Sobsey, M. D.; Erdman, D. D. Quantitative real-time PCR assays for detection of human adenoviruses and identification of serotypes 40 and 41. *Appl Environ Microbiol* **2005**, *71* (6), 3131-3136. DOI: 10.1128/AEM.71.6.3131-3136.2005.

(4) Dierssen, U.; Rehren, F.; Henke-Gendo, C.; Harste, G.; Heim, A. Rapid routine detection of enterovirus RNA in cerebrospinal fluid by a one-step real-time RT-PCR assay. *J Clin Virol* **2008**, *42* (1), 58-64. DOI: 10.1016/j.jcv.2007.11.016.

(5) Vega, E.; Barclay, L.; Gregoricus, N.; Williams, K.; Lee, D.; Vinje, J. Novel surveillance network for norovirus gastroenteritis outbreaks, United States. *Emerg Infect Dis* **2011**, *17* (8), 1389-1395. DOI: 10.3201/eid1708.101837.

(6) Turgeon, N.; Toulouse, M. J.; Martel, B.; Moineau, S.; Duchaine, C. Comparison of Five Bacteriophages as Models for Viral Aerosol Studies. *Applied and Environmental Microbiology* **2014**, *80* (14), 4242-4250. DOI: 10.1128/Aem.00767-14.

(7) Chahal, C.; van den Akker, B.; Young, F.; Franco, C.; Blackbeard, J.; Monis, P. Pathogen and Particle Associations in Wastewater: Significance and Implications for Treatment and Disinfection Processes. *Adv Appl Microbiol* **2016**, *97*, 63-119. DOI: 10.1016/bs.aambs.2016.08.001 From NLM Medline.
